# What prompts patients to present with delirium?

**DOI:** 10.1007/s41999-020-00443-7

**Published:** 2021-02-05

**Authors:** Kate Gibb, Anastasia Krywonos, Runil Shah, Anjali Jha, Daniel Davis

**Affiliations:** 1 Acute Medical Unit, University College London Hospitals NHS Foundation Trust, 1-19 Torrington Place, London, WC1E 7HB UK; 2 University College London Medical School, London, UK; 3 Department of Population Science and Experimental Medicine, MRC Unit for Lifelong Health and Ageing at University College London, London, UK

**Keywords:** Delirium, Recognition, Response, Awareness

## Abstract

**Purpose:**

To explore the recognition, response and understanding of delirium in families and carers of hospitalised patients.

**Methods:**

All adults with delirium admitted to an acute medical unit were included. Delirium was diagnosed by a specialist geriatrician. The responder who sought medical advice for each patient was interviewed using a delirium recognition questionnaire. Vital status was ascertained at four months.

**Results:**

Sixty patients were included (mean age 85, SD 6.8 years). Reported symptoms included drowsiness and lack of responsiveness, though these were less commonly recognised as being due to delirium. 76% received medical advice within 24 h, although two responders took > 1 week. One-third of responders had never heard of delirium. Delirium knowledge among responders was variable.

**Conclusion:**

Overall awareness and knowledge of delirium was poor. Community delirium education and public health initiatives may improve rapidity of recognition, delirium assessment, and potentially health outcomes.

## Key summary points

**Aim:**

To explore the public awareness and understanding of delirium and factors that prompt admission in patients presenting to hospital with delirium.

**Findings:**

Individuals responded to delirium due to a variety of symptoms and tolerated these symptoms over a range of times (from hours to weeks) before seeking medical help. Most patients received medical advice within 24 h of an identified change in state, although responders’ understanding of the change and general delirium awareness and knowledge was poor.

**Message:**

Targeted delirium education and public awareness may be warranted to improve timely delirium care.

## Introduction

Delirium, characterised by an acute disturbance of cognition, arousal and inattention, affects around 1 in 4 older inpatients [1]. It is substantially underdiagnosed, in part due to its fluctuating nature and diversity of clinical manifestations. Knowledge of patients’ baseline cognition is critical for delirium detection, and can be a barrier for health professionals if this is lacking [2]. Symptoms of delirium may be misattributed to causes, such as depression, dementia or normal ageing [3]. Hypoactive delirium is most commonly missed and is associated with worse outcomes [4]. Missed diagnoses may contribute to excess mortality [5, 6], making systematic detection of delirium essential in any setting.

Families and carers are generally well placed to identify changes in an older person’s cognitive state and they too can under-appreciate delirium symptoms if the term or concept is unfamiliar. Yet this initial recognition and response to delirium is essential to prompt medical assessment and treatment, and perhaps reduce associated morbidity and mortality. To understand this, we investigated the awareness, recognition and response to delirium symptoms in adults admitted to an acute medical unit.

## Methods

### Participants

We included adults with delirium admitted from the community to the acute medical unit at a large university hospital over four months in early 2019. Patients were approached on a convenience basis and excluded if they were under the care of another primary team (e.g. surgery, haematology, oncology). Patients who developed delirium during admission, or who were admitted from another hospital or rehabilitation unit were excluded. All data were collected by a team (KG, AK, RS and AJ) who received standardised training by KG.

### Diagnostic measures

Delirium diagnoses were made by the consultant geriatrician in charge of that patient’s care and diagnoses were confirmed by KG using a protocol which included the 4AT [7].

### Outcome measures

A delirium recognition questionnaire (Appendix 1) was developed by a clinical research fellow (KG) and a consultant geriatrician (DD). It comprised three sections: (1) process of delirium detection by health professionals; (2) recognition of delirium by the person who sought medical help, termed the responder (which may have been the patient themselves); (3) responder knowledge of delirium.

We determined vital status at four months through chart review. Deaths occurring outside of hospital were captured through daily updates on the NHS Spine, a collection of local and national databases and systems containing demographic information.

### Other variables

We recorded basic demographic and clinical information. Cognitive status was classified as: dementia, if they had a clear diagnosis on GP records or previous hospital documents; undiagnosed cognitive impairment, if there was evidence of cognitive impairment on previous records or collateral history but no formal diagnosis of dementia; or no cognitive impairment. We noted the timing of any documentation of delirium, along with any associated symptoms. The responder was identified from paramedic notes and physician history, and they were approached either in person or by phone if not available.

### Data analysis

Data were mainly descriptive, though reported symptoms were categorised in relation to length of time to response (< 6 h, 6–24 h, 1–2 days, 3–7 days, 1–2 weeks). Symptoms documented by health professionals were recorded by day of admission (day 1, 2, 3, 4 or later) as well as diagnosing service (emergency department, acute medicine, specialist geriatrician). For the symptoms used to recognise delirium, we assessed differences between health professional and responder recognition using McNemar’s test.

## Results

### Participant characteristics

On each day, data were collected, all eligible patients were included. Sixty patients were included (mean age 85 years, SD 6.77). There were 27% (*n* = 16) living with dementia, 30% (*n* = 18) with undiagnosed cognitive impairment, and 43% (*n* = 26) with no cognitive impairment.

### Delirium recognition

**Fig. 1:**
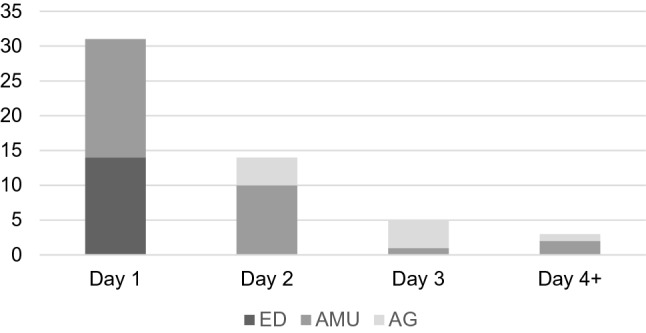
The documentation of delirium by day and team. *ED* Emergency Department, *AMU* Acute Medical Unit, *AG* acute geriatrics

Delirium was documented in the notes in 88% of cases, mostly on Day 1 by the acute medical team (Fig. 1).

### The responders

Most (63%) responders were family members; 10% were paid carers, three were friends, one neighbour, one health professional and in one case, the patient phoned the ambulance. In 15%, we were unable to identify the responder. Responders were either contacted by phone (39%) or face-to-face (37%), and 24% were unable to be contacted after three attempts. A total of 37 responders were available to complete the questionnaire.

### Responder responses

Common themes for the first indication of illness were: lack of responsiveness, drowsiness, poor appetite, confused speech, and loss of mobility or fall (Table 1). Thirteen (35%) responders reported feeling concerned, but did not know the cause for the patient’s symptoms, 7 thought the patient had an infection, 6 thought they had had a stroke, 9 thought there was another cause and 2 responders reported recognising a change, but not being concerned.

**Table 1:** First change in patient noticed, according to time taken to seek medical advice

Time to seek medical advice	First change noticed
< 6 h	"Not responding, mumbling, shaking"
	"Not eating much. Counting out loud"
	"Found her on fall"
	"Slumped to right side. Couldn't get up. Looked unwell"
	Fall. Very sensitive to pain. "Banging and shouting"
	"Took a while to answer the phone. Not himself and rambling"
	"Confused, seeing things, trying to grab things, falling asleep"
	"Kept sleeping all the time. Not eating"
	"Thinking that people were in her flat"
	Confused
	Respondent found patient shouting "help" out the window in the middle of the night
	He couldn't walk or stand up and seeing animals with lights, and rats and insects in the shower
	Collapse, shaking. Scared her daughter
	"He fell off the sofa at 4am and urinated himself. I was up with him all night. He was pulling on my wrist"
	"Very confused on the phone, starting sentences and stopping mid sentences forgetting what she was talking about. She just wasn't herself"
	Confused speech—"What is that man/ baby doing there?". She couldn't sleep at night. She came out of the toilet, fell and hit her head
	"Didn't eat his dinner", "Had a fall
	"Kept turning hot, then cold then shivering"
	"Unconscious"
	Blood shot eye and complaining of blindness in eye
	Unresponsive, confused
	Slurred speech, confused and disorientated
6–24 h	She was "Staring out the window. Stopped sleeping"
	"Restless and agitated in her sleep. Crackly in chest."
	Cold
	"His breathing was wrong, his legs were swelling and he was unable to talk"
	"She had swollen ankles and then had a fall"
	She wasn't eating or drinking
1–2 days	"Wasn’t talking properly. Slurred"
	"She went off her food"
3–7 days	When son phoned her and she said she had chest infection. 1 day later she fell out of bed
	"Kept falling forwards when sitting." Not understanding what she was saying
	"Staring. Talking to the ceiling. He did not recognise me."
	He had a fall
	"He lost his mobility, couldn't coordinate himself, wouldn't sit on the toilet seat. I had to help"
1–2 weeks	"Not eating and drinking properly"
	"Confused, very tired"

The initial response of most responders was to seek medical advice: 32% phoned an ambulance and 27% phoned the urgent help line or the general practitioner. 22% did nothing and three commented, “She was managing okay”, “I was just hoping it would get better" and "She seemed okay and would recover".

**Fig. 2:**
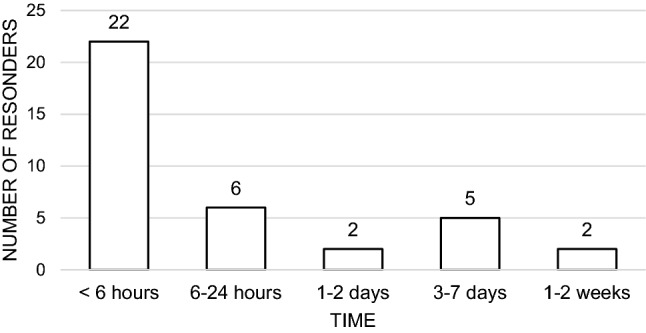
Time to seek medical advice

Twenty-two (60%) sought medical advice within six hours of the initial change in the patient, and 76% within 24 h (Fig. 2). Two responders took 1–2 weeks to respond, commenting "Very tired, confused. Just old age” and “Not eating or drinking properly” (Table 2).

**Table 2:** Response of 1–2 week responders

	First change noticed	Initial thought of responder	Initial response of responder	Reason to seek medical advice
Patient 9	“Confused, very tired”	Not concerned -"just old age"	Do nothing—"She was managing at home"	"Brother visited and said that this wasn't just old age decline"
Patient 26	“Not eating and drinking properly”	Concerned but didn’t know why	Other- “Encouraged her to drink”	Found her on floor

### Knowledge of delirium

Twenty-five (68%) responders had heard the term delirium. However, their understanding of delirium was variable (Table 3).

**Table 3:** Responder's knowledge of delirium

No knowledge	Some knowledge	Fairly accurate knowledge
“Nothing to be honest”	“They are not really normal, either the conversation or the look”	“Confusion. Can't make out what’s happened around you”
“Don’t know”	“Not quite in the real world”	“Confusion”
“I’ve heard the word but I'm not sure what it means”	“Something that we can get when we get infection or high temperatures”	“The modern term for acute confusional state”
“Nothing”	“Every time he's been admitted. Can be worse than dementia. The decline has been so rapid”	“Talking with no sense, confusion”
	“Temporary losing your mind”	
	“Someone can't make sense of what’s going on. How my father gets”	
	“All mixed up, don't know where they are”	
	“Agitated. Against me. Paranoid.”	
	“When an infection can make you confused and a bit doo-lally”	
	“When someone is behaving totally out of character and not themselves”	
	“To not know what is going on”	
	“You get it from a mini-stroke”	
	“Different from confusion. Usually accompanied with fever. Talking rubbish”	
	“Not quite right in the head. Seeing things that aren't there”	
	“An after effect of illness”, “comes across similar to Alzheimer’s”	
	“Feverish, in a confused state”, “Not quite all there”	
	“See things, talk different”	

### Responder and healthcare professional-reported symptoms

Compared with healthcare professionals, responders were less likely to recognise drowsiness, agitation or hallucinations as being part of the delirium symptoms of in the index presentation (Table 4). However, responders did consider confusion and decreased mobility as delirium symptoms.

**Table 4:** Responder and healthcare professional-reported symptoms

	Health professionals		Responder		*P*
	Number	Percentage (%)	Number	Percentage (%)	
New or worsening confusion?	55	92	34	57	0.48
Uncharacteristic drowsiness	20	33	21	35	0.06
Agitation, aggression, violence	19	32	18	30	0.1
Hallucinations	13	22	16	27	0.05
Change in mobility	31	52	26	43	0.42

### Follow-up outcomes

At follow-up, 20% (12/60) of patients had died (mean age 86 years, SD 4.87). In seven, the responder was unable to be contacted. Among the five deceased patients whose responder did complete the questionnaire, four had uncharacteristic drowsiness as identified by the responder, compared with 33% of the presentations as a whole. All responders sought medical advice within 2 days (three within 6 h), and four responders had heard of delirium.

## Discussion

We found that while family and carers recognised symptoms of delirium, their interpretation and response to them were variable. Most responders sought advice promptly, however, some took considerably longer despite identifying symptoms that gave them concern. A third of responders could either not be identified or contacted which represents a significant barrier for physicians to collate a clear account of events leading to admission. Taken together, these results suggest targeting poor awareness and understanding of delirium among families and carers could improve more prompt recognition and management.

Our data are limited to an urban population at one hospital, which may not generalise to other settings. Socioeconomic status and ethnicity of the responder were not recorded, nor were previous episodes of delirium, which may have impacted on responder behaviour. It is possible that some cases of delirium may have been missed due to reliance on ward round lists and the duty consultant to identify delirious patients. However, our study was able to capture a typical population presenting to acute care and we were systematic in our approach to standardising data collection.

Few other studies have previously examined the pre-hospital appreciation and response of delirium in patients. The awareness and recognition of delirium among family and caregivers has been reported to be low when presenting theoretical scenarios [8]. Others report that nearly 97% of family caregivers had not heard of delirium [9]. Family educational interventions have tried to improve prevention and early recognition of delirium during hospital admission [10, 11]. Such strategies could be broadened to include families’ future recognition of delirium.

Our findings highlight a need for delirium education in family and carers and for greater public awareness. Uncharacteristic drowsiness appeared to be more common in people that died within four months, suggesting this could be a particular feature worth targeting. Memory clinics are an opportunity to offer delirium education to family and carers of at-risk patients. Other potential routes of education could include local pharmacies, care staff agency, nursing home staff, paramedics and online training for health professionals. Public health initiatives, such as a delirium equivalent of the FAST test for stroke, may increase delirium awareness and response [12]. A key research priority is to investigate the time of symptom onset and its relationship to the time to medical intervention and outcomes. All education interventions would benefit from an emphasis on the recognition of hypoactive delirium with the aim of improving recognition and outcome.

## Data Availability

On request.

## Appendix

1. Delirium recognition questionnaire.
2. Responders answers for prompt to seek medical advice.

## Appendix 1: Delirium Recognition Questionnaire

Part 1

Part 2

Part 3

“We think *patient’s name* has delirium. Older people very commonly present to hospital with delirium. I describe it being like a mini-dementia. Delirium is a sudden change in brain function which usually develops over a few hours or days. People become confused, disorientated and have difficulty concentrating and paying attention. They can be more sleepy than usual or conversely, they may be agitated and restless.

There are many causes of delirium which include infection, stroke, medication, dehydration and constipation. It is more common in older people because their brain is more fragile and vulnerable, so if they become unwell their brain is more likely to functioning properly.”

## Appendix 2

**Table:**

Prompt to seek medical advice
Concerned as not responding
Normally eats all her food. Uncharacteristic behaviour (counting out loud)
Found on fall—concerned she might be cold and have fractured something
Get her checked. Worried
Thought he had had a stroke
Found her on floor
“Every time you touched him he went 'Ow, ow, ow. Don't touch me'. Slanting to the side”
Incoherent speech. Not able to get her back into bed
Concerned by different behaviour
Slurred speech returned. "Not eating"
"When he wasn't responding"
Very drowsy and not eating
Worsening confusion. Talking to the ceiling. Not responding to her
Wouldn't take her tablets—"She couldn't understand how to coordinate taking her tablets."
Her sugar level did not respond to giving her sugar
"Saying people were in her flat trying to ro her". "Emptying her drawers out"
"Brother visited and said that this wasn't just old age deterioration"
"Didn't feel comfortable leaving her. Better supervised in hospital"
Change in mental state and unable to move legs
Collapse
"Couldn't cope with him at home. He was incontinent"
"She wasn’t herself. She is normally very capable and fiercely independent"
Wanted to know why they were cold
"He was in bed delirious. He couldn't lie straight and was in total distress"
"I know when he is unwell"
"She kept going to the toilet at night and fidgeting in bed"
"Couldn't move", "Unresponsive—stares through me"
"He wasn’t lying straight in bed, he just wasn't right"
Saw blood in urine when she went to bathroom
"Something like a UTI can become dangerous—like urospesis in someone her age, which can be life-threatening"
His brother who is a paramedic suggested to call 999 as he was worried about a stroke
" I thought it was a UTI but ankles had never been this swollen before"
"She was not acting herself, hadn't eaten or drunken in a while and wasn't speaking"
Concerned about bloodshot eye
"It was scary and longer than acceptable"—confusion lasted longer than was normal
Very confused—worried she had had a stroke

## Code availability

On request.
